# Supplementary material for: Mutations in PCYT2 disrupt etherlipid biosynthesis and cause a complex hereditary spastic paraplegia
Source: Brain. 2019 Oct 22;142(11):3382–97. doi: 10.1093/brain/awz291 (PMC6821184; doi:10.1093/brain/awz291)
Supplement: awz291_Supplementary_Data [file awz291_supplementary_data.zip › awz291-Suppl_data/Supplementary_Data4.zip!/Supplementary information 4 - plasma lipidomics.zip!/Plasma lipidomics/Control vs Patient/Statistical plots and data/S1P species/Boxplots.pdf]

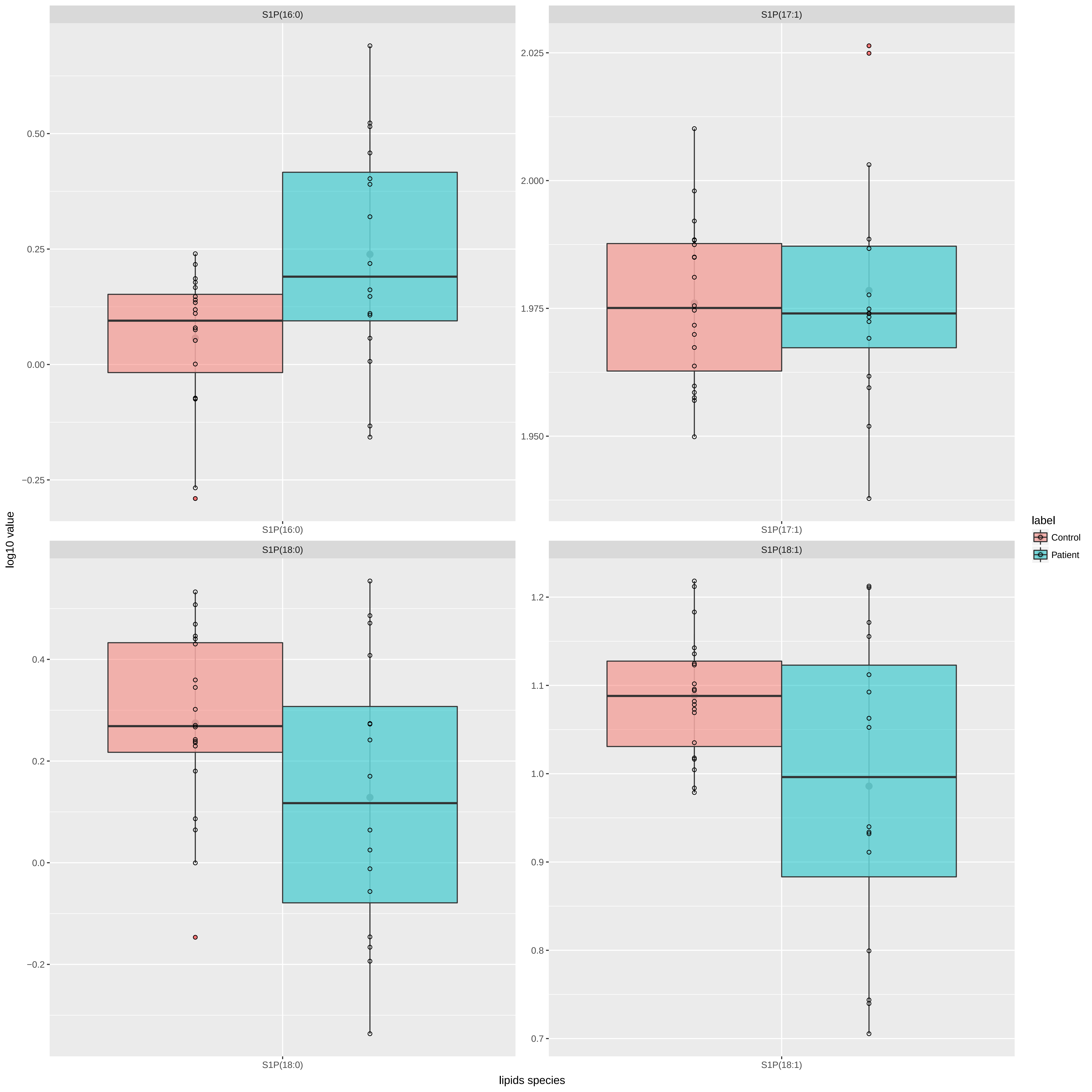

S1P(16:0)
S1P(17:1)
S1P(18:0)
S1P(18:1)
0.50
0.25
0.00
−0.25
2.025
2.000
1.975
1.950
0.4
0.2
0.0
−0.2
1.2
1.1
1.0
0.9
0.8
0.7
log10 value
lipids species
label
Control
Patient
